# VARIATION IN USE OF HOSPICE AIDES BY RESIDENTIAL SETTING

**DOI:** 10.1093/geroni/igad104.1115

**Published:** 2023-12-21

**Authors:** Jennifer Reckrey, Karen Mckendrick, Melissa Aldridge

**Affiliations:** Icahn School of Medicine at Mount Sinai, New York City, New York, United States; Icahn School of Medicine at Mount Sinai, New York City, New York, United States; Icahn School of Medicine at Mount Sinai, New York City, New York, United States

## Abstract

Hospice care frequently includes visits from hospice aides, but need for hospice aide care may be different for those in the community compared to settings like assisted living or nursing homes where personal care is routinely provided. The objective of this longitudinal cohort study is to identify factors associated with hospice aide visits (any versus none) and frequency of hospice aide visits (proportion of routine hospice cays with an aide visit) in different residential settings (community, assisted living, and nursing home). We identified Medicare beneficiaries enrolled in the Medicare Current Beneficiary Survey (MCBS) who died between 2010-2019 (n=1,915). We used self-reported survey responses, Medicare claims data, and Medicare Provider of Service data to compare decedent hospice care experience across residential settings and estimated multivariable models to compare factors associated with hospice aide visits among residential settings. Hospice aide visits were least common in the community setting (64.4% vs 76.6% vs 72.6% with any hospice aide visits in community, assisted living, and nursing home respectively, p=0.002). In models adjusted for sociodemographic, clinical/functional, and hospice factors, neither hospice aide visits nor the proportion of routine hospice days with hospice aide visits varied significantly by residential settings. Future work to understand when and why hospice aides are utilized and how hospice aides collaborate with families and existing care teams in will help ensure that hospice care is appropriately tailored to individual care needs in all residential settings.

